# Factors Facilitating and Inhibiting the Implementation of Telerehabilitation—A Scoping Review

**DOI:** 10.3390/healthcare12060619

**Published:** 2024-03-08

**Authors:** Susanne Stampa, Christine Thienel, Pinar Tokgöz, Oliver Razum, Christoph Dockweiler

**Affiliations:** 1 Department Digital Health Sciences and Biomedicine, School of Life Sciences, University of Siegen, 57076 Siegen, Germany; 2 School of Public Health, Bielefeld University, 33615 Bielefeld, Germany

**Keywords:** telerehabilitation, implementation science, barriers, facilitators, Consolidated Framework for Implementation Research

## Abstract

Due to the coronavirus pandemic, telerehabilitation has become increasingly important worldwide. While the effectiveness of telerehabilitation is considered proven for many indications, there is comparatively little knowledge about the implementation conditions. Therefore, this scoping review summarises the current state of facilitating and inhibiting factors that may influence the uptake of telerehabilitation. The review follows the JBI methodology for scoping reviews. The article search was carried out in five databases (MEDLINE, EMBASE, Web of Science, Cochrane and Psyndex) in May 2022, with an update in October 2023. Two independent researchers identified relevant studies according to the inclusion and exclusion criteria. The Consolidated Framework for Implementation Research served as the theoretical basis for the categorisation of the facilitating and inhibiting criteria in the organisational context. A total of 28 studies (timespan 2012 to 2023) have been included. The most relevant barriers identified are technical issues and a lack of technical skills. The factors considered most favourable for implementation are patients’ motivation and the involvement of high-level leaders. The results provide clear indications of factors that inhibit and facilitate implementation, but also show that further research is needed.

## 1. Introduction

Telerehabilitation (TR) has become increasingly important worldwide in recent years [1]. A key driver was the coronavirus pandemic, which led to massive restrictions regarding personal contact. During this period, the World Health Organization recommended the postponement of treatments that were not considered urgent in order to ensure safety and still guarantee essential rehabilitation services [2].

Consequently, to meet hygiene requirements and minimise the risk of infection, rehabilitation centres were challenged to find new ways to deliver and maintain treatment or aftercare [3]. As part of this process, a variety of TR offers were developed to enable rehabilitation patients to continue treatment in their home environment [4,5].

In the context of this scoping review, TR refers to the provision of medical rehabilitation services using information and communication technologies (ICT). These rehabilitation services can be offered across existing geographical and/or temporal distances [6,7]. They encompass a range of rehabilitation services, including monitoring, prevention, intervention, consultation and counselling, and can be provided by many health professionals (e.g., physiotherapists, speech pathologists, occupational therapists). TR addresses both children and adults in various settings and with different medical conditions [7].

Beyond the pandemic situation, TR has the potential to close gaps in care and provide rehabilitation services, regardless of time and place. This is also advantageous for rehabilitation patients who, for example, live in structurally weak and rural areas or have limited access to rehabilitation facilities due to immobility and/or multimorbidity [8].

Numerous studies and reviews confirm the effectiveness of TR for physical disabilities, as well as mental disorders, in many settings [9,10,11,12]. However, the situation is different for the implementation processes of TR. As there was an urgent need to create alternative services quickly during the coronavirus pandemic, there was hardly any time to develop implementation concepts and conduct implementation research [13]. Many rehabilitation centres that have introduced digital services have not used implementation strategies or have taken an unsystematic approach to their introduction [14]. Our scoping review, therefore, aims to analyse the current state of research on the implementation conditions of TR in order to identify barriers and facilitators for implementation. Implementation frameworks enable a systematic assessment of these factors and, thus, help to understand the complexity of the implementation processes. In particular, the Consolidated Framework for Implementation Research (CFIR) seems to be suitable, as it can be seen as a synthesis of existing implementation theories [15].

To the best of our knowledge, there is no review of barriers and facilitators in the implementation of TR that takes into account all indication groups, as well as all types of TR technologies (e.g., apps, videoconferencing programmes or virtual reality), and systematically maps them on the basis of an implementation theory. However, during the course of the literature search, systematic and scoping reviews were identified that overlap with the topic of our study. These reviews either focus on a specific indication area or a broader thematic focus that does not explicitly concentrate on factors that hinder or promote implementation. In addition, the publications are often based on a broader understanding of implementation, which also includes studies that focus exclusively on the use and acceptance of TR [14,16,17,18,19]. In contrast, this scoping review is based on an understanding of implementation that considers the implementation process of TR as a process involving different organisational levels and stakeholder groups [15]. It provides a broad overview of existing research on the conditions for the implementation of TR and focuses on studies conducted before, during and after implementation. Therefore, the scoping review answers the following research question:

What barriers and facilitating factors can be identified for implementing TR in medical rehabilitation across all indication groups and TR technologies?

## 2. Materials and Methods

A scoping review was chosen to address the complexity of the research question and provide a broad overview of the current state of research. The scoping review was pre-registered with Open Science Framework 25 May 2022 (https://osf.io/saqyu, accessed on 11 January 2024) [20]. The methodology of the scoping review followed the methodological approach described by Elm et al. (based on the JBI methodology), as well as the framework for scoping reviews developed by Arksey and O’Malley [21,22,23]. To ensure transparency, the PRISMA extension for Scoping Reviews (PRISMA-ScR) was also taken into account [24].

### 2.1. Inclusion Criteria

Using the JBI framework principles of population, concept and context (PCC), the review was based on the following inclusion criteria [22]:

Population: All indication groups of rehabilitation were included in the review. There was no age restriction for the participants.

Concept: The scoping review is based on a broad understanding of the concept of TR, encompassing all information and communication technologies, including virtual reality (VR). It also explicitly refers to entire implementation processes. If the sole focus of the studies was the usability or acceptance of TR, the articles were excluded. If acceptance or usability was part of the investigation of the described implementation process and/or an implementation theory was used as a basis, the article was included.

Context: The scoping review included all studies on facilitating and inhibiting factors for the implementation of TR in outpatient and inpatient settings, regardless of the region or country in which they were conducted.

Types of literature: In order to obtain a broad overview of the topic, all types of studies published from 2012 to 2023 were considered, regardless of their methodological quality. Only study protocols, abstracts of conference papers and discussion papers were excluded, as well as studies not written in English or German. Reviews (e.g., systematic reviews or scoping reviews) were not included, but if they at least partially addressed barriers to or facilitators of TR implementation, they were investigated for relevant studies.

### 2.2. Search Strategy

The search strategy for the scoping review was carried out in three steps [22]. Firstly, a comprehensive search string was developed in MEDLINE as a basis for further database searches, including all terms relevant to the research question and appropriate synonyms. This was tested and adapted to search other databases, taking into account database-specific characteristics (see Supplementary Materials). The final search was carried out in May 2022 by two researchers (SSt and CT) in MEDLINE, EMBASE, Web of Science, Cochrane and Psyndex. In order to keep the results as up to date as possible, an update was carried out in October 2023. In a third step, the reference lists of all studies which met the inclusion criteria (including the reviews found) were searched, as well as the “grey literature”, which was searched using Google Scholar to include publications that had not been published in the usual databases. Table 1 shows an example search string for the Cochrane database.

### 2.3. Study Selection

**Figure 1:**
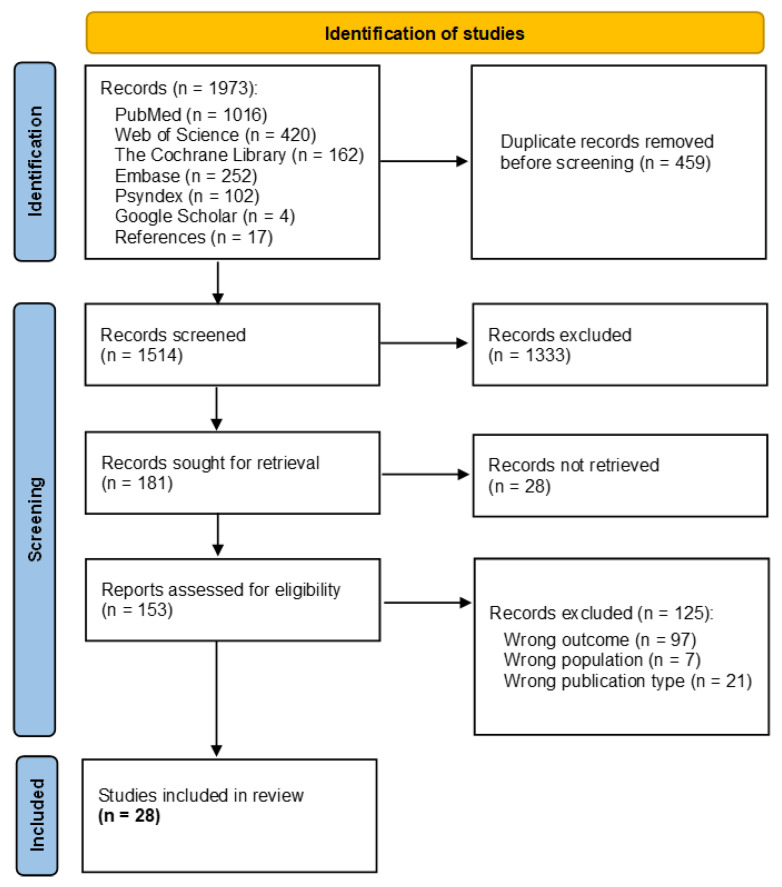
Selection process using the PRISMA flow diagram.

The search resulted in a total of 1973 hits, which were imported into the Rayyan 2022 software. After removal of duplicates (*n* = 459), two authors (SSt and CT) independently screened the titles and abstracts of the remaining articles for relevance and removed articles that did not meet the inclusion criteria. Disagreements regarding the inclusion or exclusion of an article were resolved in discussions after unblinding. This left 153 full-texts, which were also reviewed independently for their relevance to answering the research question. Where discrepancies arose, these were clarified through further discussion. In addition, the authors checked the reference lists of the included studies and the reviews found in order to identify further relevant articles. Finally, 28 studies were included. Figure 1 shows the process of study selection using a PRISMA flowchart [24].

### 2.4. Analysing the Data

In order to structure the results, an Excel spreadsheet was developed in which we extracted the metadata as follows: first author, year, country, study design, study population (patients), study population (health professionals), diagnosis group, type of TR, implementation status/experiences with TR, and implementation framework. However, due to the large scope of the studies, the factors facilitating and inhibiting the implementation of TR were coded using MAXQDA 24 software. This allowed for direct mapping to the CFIR domains and constructs. The data were extracted from the articles independently by the two authors SSt and CT. Discrepancies were discussed within the team.

## 3. Results

### 3.1. Study Characeristics

All 28 included studies were published between 2014 and 2023, and all but three studies [25,26,27] were conducted in high- or upper-middle-income countries [28]. Most of these (*n* = 10) were undertaken in Canada [29,30,31,32,33,34,35,36,37,38], followed by 5 studies in Australia [39,40,41,42,43] and 4 studies in the USA [44,45,46,47]. The three studies conducted in lower-middle-income countries were from India [25], Iran [26] and Uganda [27].

Of the 28 included publications, 17 addressed the research question using a qualitative design [26,29,32,33,34,35,36,37,39,42,43,44,45,46,47,48,49], while 5 studies worked with a quantitative design [25,40,50,51,52] and 5 studies were based on a mixed methods approach [27,30,38,41,53]. One of the included articles was a synthesis of six qualitative and quantitative studies with different study designs, which were mentioned, but not explained in detail (pre-post, pilot studies, RCTs, observational designs) [31]. With the exception of one study, where a cognitive work analysis was conducted [34], all qualitative research was based on interviews and/or focus groups. The quantitative studies were exclusively online surveys, and the mixed methods studies used quantitative surveys [30,38] as well as interviews and focus groups [27,41], each supplemented by other data sources (process data, e-mail correspondence, etc.). One of the mixed-methods studies was a combination of a survey and focus groups [53]. The majority of studies (*n* = 16) were cross-sectional in design [25,26,29,32,36,37,39,42,43,44,46,48,50,51,52,53], followed by pre-and post-design [30,38,40,47,49]. In qualitative studies, the sample sizes ranged from *n* = 3 to *n* = 26, and in quantitative studies, they ranged from *n* = 26 to *n* = 513. The detailed characteristics of the included studies can be found in Supplementary Materials.

### 3.2. TR Technologies

The TR programme described in the included studies consisted largely (*n* = 15) of therapies that were delivered via videoconferencing programmes, using laptops, PCs and tablets [29,31,32,34,35,38,40,42,44,46,47,48,50,52,53]. Some of these were also offered as group therapy [38,40,42]. Furthermore, gaming software (e.g., Nintendo Wii^©^, WiiFit^©^) was applied in two studies to promote the recovery of physical functions [30,31]. Three studies used sensor-based technologies (wearables) to track physical activity [34,37,50], as well as VR-technologies [30,31,50]. Other complementary digital offers were exercise videos [32,41,45], e-learning modules [34,35], emails [46], SMS [27] and phone calls [27,33]. In four studies TR was not described in more detail [26,36,39,43].

### 3.3. Population and Indication Groups

In 19 studies, TR was analysed exclusively from the perspectives of healthcare professionals [26,27,29,30,32,36,37,39,40,42,43,44,46,47,48,50,51,52,53], while seven studies also included the patients’ perspectives [25,31,33,34,35,41,45]. Two studies analysed only the patients’ perspectives [38,49]. The target groups of TR were generally adults, and three of them focused exclusively on older patients [37,52,53]. Only four studies explicitly treated children or involving familiy members [27,30,33,40]. The most frequently included indication group was neurological disorders [27,29,31,35,36,38,40,42,51,53], followed by orthopaedic disorders [32,37,39,40,41,53]. Five studies did not mention any explicit diagnoses [26,30,44,47,50].

### 3.4. Status of Implementation

Eleven of the included studies were conducted before the introduction of TR into routine care. Respectively, the barriers and facilitating factors were anticipated by the participants or collected, for example, as part of a feasibility study [25,27,30,31,33,39,41,42,45,49,53]. Five studies investigated inhibiting and facilitating factors during the introduction into routine care [29,34,38,40,47], and five studies looked at these aspects after the introduction of TR had already taken place [35,36,43,44,46]. Seven studies did not refer to a specific rehabilitation centre but to a specific technology and its implementation conditions, so the implementation status was measured by the experience of the participants. In two of these studies, all of the participants had experience with TR [26,32], and in five studies, some of the participants had previous experience, whereas others had no experience [37,48,50,51,52].

### 3.5. Implementation Frameworks

Ten of the included studies were based on an implementation framework [27,29,37,38,40,45,46,47,48,51], and four used other theoretical frameworks [30,31,34,35]. The CFIR [29,45,46], the Theoretical Domains Framework (TDF) [40,45,48] and the Reach, Effectiveness, Adoption, Implementation, Maintenance Framework (RE-AIM) [38,47] were used most frequently. Occasionally, the implementation conditions reported in the studies referred to the Promoting Action on Research Implementation in Health Services Framework (i-PARIHS) [27], the Medical Research Council Framework (MRC) [37] and the Framework of Grol [51].

### 3.6. Barriers and Facilitators for Implementation of TR

The barriers and facilitating factors for the implementation of TR are illustrated using the CFIR. The CFIR is a synthesis of various implementation theories and is one of the most frequently used frameworks in implementation science. Its overarching goal is to predict or explain factors that inhibit or promote the implementation of a technological innovation. Therefore, it can be used before, during and after implementation [54]. The five main domains of CFIR (innovation, internal setting, external setting, individuals and implementation process) with their comprehensive substructures map the entire implementation process and interact with each other. Due to the complexity of the framework, not all domains and constructs need to be displayed in the implementation processes [15]. Nevertheless, the barriers and facilitating implementation factors extracted from the 28 admitted studies represent the majority of the CFIR domains.

A total of 75 barriers and 60 facilitating factors were identified. The complete list of all factors, categorised according to the CFIR domains, can be found in Table 2. As shown, certain aspects can both promote and hinder implementation.

On the staff side, technical issues and limited knowledge about the innovation were cited as the most common barriers to implementation. Technical barriers included, for example, general problems with technical devices, the initial setup and distribution of apps or platforms and poor video quality [26,27,30,34,38,41,42,43,44,46,48,50]. Limited staff knowledge was related to the handling of the technological innovation and the implementation process itself [25,26,31,32,33,36,37,39,40,41,45,46,50,52].

Many of the staff members also stated a lack of time [25,29,30,33,34,37,38,41,52] and a lack of financial resources [25,33,34,38,39,42,46,50,52] to implement and maintain TR. Other important mentioned barriers were a lack of personnel [25,26,33,34,35,50], limited treatment options [26,29,32,43,46,47,48], the poor health statuses of patients [26,29,37,38,41,49], external policies [25,45,46,47,50] and the adaptability of the TR service [29,33,41,46,52].

With regard to the patients, a lack of technical skills [25,29,32,34,36,39,45,46,53], a lack of acceptance [25,26,32,37,46,47] and a lack of technical equipment or internet access [25,29,32,33,36,39,42,46,48,49] were often named as inhibiting factors.

Apart from the technical difficulties encountered at any stage of implementation, the greatest barriers described were mostly found in studies where TR was not yet part of routine care. For example, a lack of staff skills is mostly described before the implementation of TR [25,31,33,39,41,45] or mentioned in studies where some of the participants have worked with it [32,37,50,52]. Time and financial resources are barriers that are also mostly identified before implementation [25,30,33,39,41,42] and during the implementation process [29,34,38]. These barriers were also described by people who already had experience with TR [26,37,50,52] but only mentioned in one study that had already transferred TR into routine care [46]. The same applies to patients’ technical skills [46], although Munce et al. also recognised this problem in elderly and multimorbid patients after the introduction of TR [36]. Other barriers, such as the adaptability of the TR platform or limited treatment options, affect all levels of implementation and experience.

Factors that facilitate implementation are mainly related to the involvement of programme leaders [29,33,35,36,43,45,46,47], as well as to the attractiveness of the TR programme for patients. These include benefits such as independence of location, less travelling, shorter waiting times, and the independent continuation of therapy [25,31,32,46,47,48,51]. Other important facilitators are patient motivation and compliance [27,30,31,37,39,43,51,53], staff training [31,36,39,42,43,46,48], support with any technical problems and the introduction of the new service [36,42,43,44,46,51,52] and the ease of use of the programme [29,31,42,43,51,52].

Looking at the facilitators of TR according to the stage of implementation, the most frequently mentioned aspect, the involvement of programme leaders, is mainly named in studies that look at implementation retrospectively [35,36,43,46] or are in the implementation phase [29,47]. Only two studies describe this facilitator prospectively [33,45]. Technical support is only considered beneficial in one pre-implementation study [42]. On the other hand, the facilitator of patient motivation was mainly described before implementation [27,30,31,53], while staff training is seen as helpful by many participants before and after implementation in routine care [31,36,39,42,43,46]. Ease of use was mentioned at all stages of TR uptake and all levels of experience.

## 4. Discussion

There are many studies that have investigated the implementation conditions of TR. Also, some scoping reviews and systematic reviews were found. However, none of the papers provided a systematic overview of inhibiting and facilitating factors that influence the implementation of TR across all indications and technologies, using an implementation framework. Our search identified 28 studies that met the inclusion criteria. When selecting the studies, particular attention was paid to the differentiation between factors that promote and inhibit the implementation of TR and factors that influence the use and acceptance of TR. For this reason, studies that postulated similar barriers and facilitating factors, but did not explicitly relate these to the implementation process, were excluded. This distinguishes our review from comparative reviews, which are often based on a broader definition of implementation [17,19].

In our review, the factors influencing implementation are presented within a broad theoretical framework that provides different starting points for addressing barriers and facilitating factors for successful implementation [15,54]. Most of the barriers, as well as the facilitating factors, are found within the TR centres themselves, thus addressing the domain *Inner Setting* of the CFIR. This relates, in particular, to technological issues, as well as spatial, time, financial and personnel resources. It is also mentioned in the scoping reviews by Pearce et al., who analysed current strategies to support and evaluate the implementation of TR; Glegg & Levac who investigated inhibiting and facilitating factors for the implementation of VR in rehabilitation; and Nizeyimana et al., who mapped the feasibility, cost and access to TR [14,16,17].

Ross et al. and Pitt et al. looked for solutions to these barriers and suggested that technical problems could be solved with the help of tech-savvy colleagues or additional staff [42,43]. This is consistent with the fact that one of the most important facilitators is technical support [36,42,43,44,46,51,52].

Other barriers and facilitators lie within the systems themselves and are based on the systems used (CFIR domain: *Innovation*). They relate to the direct delivery of rehabilitation programmes. For example, physiotherapists find it restrictive that patients can only be taught the exercises on screen, whereas important other components, such as the palpation of the patient, are omitted in the course of TR [26,29,32,43,46,47,48,53]. This has been also reported in other studies [17]. Hale-Gallardo et al. and Ross et al. suggest minimising this barrier by demonstrating alternative means of palpation. These could include self-palpation or the use of other methods such as patient feedback and patient reported outcomes [43,47]. The adaptability of the technology is seen as both a challenge [29,33,41,46,52] and a facilitating factor [33,37,46] in this domain. This trend works hand in hand with the usability of the system, which is seen as crucial for successful implementation [29,31,42,43,51,52]. Nizeyimana et al. and Glegg et al. also emphasise that this is one of the most important factors favouring the introduction of new technologies [16,17]. In addition, the high attractiveness of the offer for patients promotes the implementation of TR [25,31,32,46,47,48,51]: independence of location, shorter waiting times and independent continuation of therapy are positive aspects that are also reported from neighbouring disciplines such as telehealth [55].

Some of the most serious barriers identified lie within the CFIR domain of *Individuals*. Limited knowledge of how to use the technology is often highlighted from the perspectives of both staff and patients [25,26,29,31,32,33,34,36,37,39,40,41,45,46,50,52,53] This is complemented by patient acceptance [25,26,32,37,46,47]. Both aspects are also addressed by other reviews [14,16,17]. Kraaijkamp et al. have proposed solutions to overcome these barriers. They emphasise the importance of ongoing training of health professionals and the integration of e-health content into education, as well as co-creation and behaviour change techniques, as part of an implementation strategy [52].

At the individual level, there is another factor that is particularly facilitating. Both in our study and in the works of others, it is clear that managers involved in the implementation process can act as drivers of innovation [29,33,35,36,43,45,46,47].

External factors (CFIR domain: *Outer Setting*) that affect or interact with an organisation are not considered critical but are still important. As some of the results show, political influence and restrictions can hinder the implementation of TR [25,45,46,47,50]. The role of policy in the implementation of TR has so far rarely been investigated [17]. Further studies are needed to analyse the influence of external conditions on the implementation of TR in specific contexts and countries.

Relatively few obstacles are reported in the fifth CFIR domain, *Implementation process*. Unclear processes [27,33,46], coordination difficulties [41] or a lack of support during implementation [36,41] are occasionally mentioned. Yosef et al. emphasised the need to develop guidelines to highlight the potential problems therapists face when using TR and provide tools to overcome them. These could include strategies for successful inter-disciplinary collaboration [53]. This seems significant, because interdisciplinary cooperation is seen as the most favourable factor at the level of the implementation strategy [29,44,45,48,52].

In addition, many study participants emphasised that TR should not permanently replace conventional rehabilitation, but that a hybrid of face-to-face treatment and TR could be an important factor in implementation and help to overcome some of the barriers mentioned above [29,31,32,45,46].

Another interesting finding is that the implementation factors vary depending on the implementation status, and many barriers, such as time and financial resources, occur more frequently during the implementation process or are expected by stakeholders prior to implementation. Further research is needed to categorise barriers and obstacles according to their level of implementation in order to develop appropriate strategies. However, this would exceed the scope of this review. Furthermore, this categorisation is currently still difficult and not representative, as there are not yet many studies that look at the influences of implementation from the perspective of routine care.

The barriers described should also be investigated for different sub-groups in the future, such as age and diagnosis groups. It is beyond the scope of this review to discuss this in detail, but it is worth mentioning. In the three studies that explicitly focused on older patients, age was not found to be a fundamental barrier to the use of TR, although there were some challenges, such as technical difficulties [37,52,53]. Other possible barriers, for example, hearing or visual impairments, are also described for this population [32]. On the other hand, the elderly are also said to be open to TR [31,47]. This aspects should be considered in more detail in further studies. With regard to the different diagnoses, no difference were found. However, it must be taken into account that the results are not fully comparable due to the heterogeneity of the studies.

A further differentiation can be made in relation to lower-middle-income countries and middle- and higher-income countries. Our review found three studies from lower-middle-income countries that analysed the factors for implementing TR [25,26,27]. In our case, the results for facilitators and barriers do not differ essentially from higher-middle income-countries. However, this does not mean that this is not an important issue for the implementation of TR. Studies from the neighbouring field of telemedicine show that these technologies can offer lower- and middle-income countries an opportunity to close gaps in care [56]. Therefore, the implementation conditions of TR for these countries should definitely be analysed in more detail in further studies.

The findings of our study have some limitations, which need to be taken into account. When considering the results, it should be borne in mind that rehabilitation systems are structured differently or include different services and the concept of rehabilitation has different meanings in different countries and across indication groups.

Moreover the search strategy used may have led to relevant articles being omitted, and the distinction between factors influencing implementation and factors influencing use or acceptance was difficult in some cases. The authors discussed these cases and made a joint decision to include or exclude them. On another note, the independent assignment of the extracted data to the categories of barriers and facilitating factors of the CFIR may have led to results that differ from the analysis of other coders. It should also be considered that the wide range and heterogeneity of TR (for example, the use of VR and the treatment of patients via video conferencing systems) makes it difficult to compare implementation conditions. Last but not least, the poor methodological quality of some of the included studies should be mentioned. This is often due to the small sample size and applies to both qualitative and quantitative surveys.

Nevertheless, the authors were able to present and discuss barriers and facilitating factors for the implementation of TR in detail. This will help all groups of stakeholders to have a better understanding of what is important in the introduction of these technologies. Further implementation research, especially through studies of higher quality and with larger sample sizes, should be conducted in order to obtain valid results regarding the factors influencing the implementation of TR services. Our aim was to gain a broad overview of the current stage of research. Consideration should also be given to conducting a systematic review, which could be methodologically more comprehensive.

## 5. Conclusions

In summary, the scoping review shows that there are both facilitating and inhibiting factors influencing the implementation of TR, particularly at the organisational and individual levels. Technical difficulties and a lack of technical skills among stakeholders need to be addressed to enable successful implementation. Support by giving technical assistance and training staff and patients should, therefore, be mandatory in implementation processes.

## Figures and Tables

**Table 1:** Search string for Cochrane (search date 18 May 2022, updated on 2 October 2023).

Number	Type of Search Term	Search Term
#1	MeSH	telerehabilitation
#2	ti,ab,kw	telerehab*
#3	ti,ab,kw	tele-rehab*
#4	ti,ab,kw	erehab*
#5	ti,ab,kw	e-rehab*
#6	ti,ab,kw	virtual* NEAR/5 rehab*
#7	ti,ab,kw	remote* NEAR/5 rehab*
#8	ti,ab,kw	digital* NEAR/5 rehab*
#9	ti,ab,kw	online NEAR/5 rehab*
#10	ti,ab,kw	mobile NEAR/5 rehab*
#11	ti,ab,kw	web-based NEAR/5 rehab*
#12	ti,ab,kw	computer-based NEAR/5 rehab*
#13	ti,ab,kw	internet-based NEAR/5 rehab*
#14	#1 OR #2 OR #3 OR #4 OR #5 OR #6 OR #7 OR #8 OR #9 OR #10 OR #11 OR #12 OR #13	
#15	ti,ab,kw	influenc*
#16	ti,ab,kw	barrier*
#17	ti,ab,kw	inhibit*
#18	ti,ab,kw	imped*
#19	ti,ab,kw	hinder*
#20	ti,ab,kw	hindrance*
#21	ti,ab,kw	facilitat*
#22	ti,ab,kw	promot*
#23	#15 OR #16 OR #17 OR #18 OR #19 OR #20 OR #21 OR #22	
#24	MeSH	diffusion of innovation
#25	ti,ab,kw	diffusion of innovation
#26	ti,ab,kw	implement*
#27	ti,ab,kw	adopt*
#28	ti,ab,kw	realis*
#29	ti,ab,kw	realiz*
#30	#24 OR #25 OR #26 OR #27 OR #28 OR #29	
#31	(#14 AND #23 AND #30) AND (publication date between May 2012 and October 2023)	

**Table 2:** Barriers to and facilitators of the implementation of TR.

CFIR Domain	Construct	Facilitators	Barriers
Innovation	Innovation Evidence Base		Quality and validity of TR programme [29,33,41,46]
			Less objectivity and specificity [29]
	Innovation Relative Advantage	Attractiveness for patients: less travelling and shorter waiting time, personalised interventions, independent continuation of therapy [25,31,32,46,47,48,51]	
		Error-free functioning of the innovation [38]	
		Access for more patients [46]	
		Greater flexibility (for both sides) [46]	
		Similar interfaces to other well-known systems [33]	
		Consistency of schedule and programme components [46]	
		Efficiency of the innovation [42]	
			Limited treatment methods, e.g., inability of palpation [26,29,32,43,46,47,48]
			Reduction in patient interaction through TR [26]
	Innovation Adaptability	Adaptability with existing programmes [33,37,46]	Platform flexibility and adaptability [29,33,41,46,52]
	Innovation Complexity	Ease of use [29,31,42,43,51,52]	Usability [27,32]
		Availability of FAQs [51]	
		Availability of video instructions [51]	
	Innovation Design		Unrealistic treatment environment [26]
	Innovation Cost		See “resources”
Inner Setting	Structural Characteristics: Physical Infrastructure	Use of existing infrastructure [45,46]	
		Adequate space, consultation rooms and equipment [36,38,43]	Limited space [36,39,47]
			Lack of specialised therapy equipment at patients’ home [26,32,36]
	Structural Characteristics: Information Technology Infrastructure		Internet failure [26,31,42,46]
			Technological issues like server breakdowns, poor video quality and initial implementation (clinicians) [26,27,30,34,38,41,42,43,44,46,48,50]
	Structural Characteristics: Work Infrastructure	Clearly defined roles and responsibilities [31]	
		Flexibility of clinical regulations, organisational policies and procedures [33]	High level of bureaucracy [26]
		Development of policies and procedures [46]	Ineffectiveness of organisational policies [25]
		Technological support [36,42,43,44,46,51,52]	Slowness of technical support [31]
			High workload [41]
	Relational Connections	Embedding in interdisciplinary care [44]	
		Positive reinforcement [25]	
		Counselling [25]	
	Communication	Supportive and effective communication strategies [30,42,43,46]	No clear communication pathways were established [46]
		Information provision about the application and processes [25,32,52]	Lack of information about the innovation and its implementation (staff) [46]
		Common language [33]	Lack of common language between patient, therapist, and technologist [26]
			Intervention not available in local language [25,27]
			Challenges in virtual communication [32,46,48]
			Maintenance of platform information [33]
	Culture: Human-Equality-Centredness	Right of co-determination (staff) [48]	
	Culture: Recipient-Centredness	Participants support and connectedness [46,47]	Safety of the patients, e.g., dysphagia or risk of falling [26,29,45,46]
		Relationship building with participants [38,46]	Unreachability of patients/relatives in vulnerable or risky situation [33]
			Patients are pushed less [48]
			Privacy concerns (patients) [26,41]
	Culture: Learning-Centredness	Exchange and problem solving of challenges [46]	
	Compatibility		Incompatibility with existing technology [42]
			Incompatibility with existing workflows [46]
			Incompatibility with psychosocial components of clinical practice [35]
			Inability to conduct group-based sessions [45]
			Lack of appropriate patients (lack of experience) [27,30]
	Relative Priority	Prioritisation of the innovation [36]	Lack of priority for TR [26]
	Incentive System		Lack of tangible or intangible benefits or incentives [46]
	Available Resources	Financial resources [52]	Lack of financial resources/financing for the programme [25,33,34,38,39,42,46,50,52]
		Time to learn [51,52]	Lack of time resources [25,29,30,33,34,37,38,41,52]
		Adequate technical resources in TR centres [29,36,43,44,46,52]	Lack of adequate technology and software in rehab centres [46,47]
		Well-trained staff [38,46]	Lack of human resources, e.g., trained staff [25,26,33,34,35,50]
			Lack of technical equipment and internet access in patients’ home [25,29,32,33,36,39,42,46,48,49]
			Insufficiency of available resources to support the innovation [26,46]
	Access to knowledge	Staff training [31,36,39,42,43,46,48]	
Outer Setting	Local Conditions	Good infrastructure to attract technical staff [47]	Lack of infrastructure [36,41,47,53]
			Location of healthcare institute [50]
			Limited internet service in rural areas [33]
	Partnership and Connections	Feedbacks from audits [38]	
		Broad stakeholder involvement [33]	Not existing networks [35,46]
		Collaboration with patients’ caregivers [53]	Poor cooperation with other stakeholders [26]
	Policies and Laws	Ministry support and guidelines [33]	External policies [25,45,46,47,50]
		Integration of the providers’ professional knowledge/mentorship [33,46,47]	Providers willingness [33,50]
			Lack of government initiatives and support [25,26]
	Financing	Insurer buy-in and payment [46]	Insurance coverage/cost assumption [25,32,46]
		Lower costs for internet providers [33]	
	External Pressure	Marketing and advertising [46]	
Individuals	High-level Leaders	Managers and programme leaders’ involvement [29,33,35,36,43,45,46,47]	Non-involvement of managers and programme leaders [33,46]
	Implementation Team Members	Structure of the team [29]	Change in team structure [29]
			Role of therapists in providing troubleshooting support for technology breakdowns [42,46]
	Other Implementation Support	Family/peer support for patients [25,33,37]	Lack of social support [25,37,40]
	Capability	Familiarity with the innovation [46]	Limited knowledge (staff) [25,26,31,32,33,37,39,40,41,45,46,50,52]
		Experiences with TR in general [35]	Bad experiences with TR/Feeling inexperienced (staff) [29,42]
			Insecurity (therapists) [48]
			Lack of awareness among health professionals [25]
			Technology competence/lack of technical skills (patients) [25,29,32,34,36,39,45,46,53]
			Lack of (digital) health literacy (patients) [25,41,45]
			Poor health status of patients [26,29,37,38,41,49]
	Opportunity		Interruptions at home (patients) [49]
			Challenges in incorporating the programme in daily routine (patients) [49]
	Motivation	Comfortability using the TR technology (staff) [38]	Negative perception of TR by therapist [42,46]
		Willingness/acceptance of therapists [43]	Lack of willingness and professional motivation (staff, provider) [33,46]
		Patients’ motivation, willingness and compliance [27,30,31,37,39,43,51,53]	Non-compliance and demotivation (patients) [25,27,32,35]
		Self-efficacy/determination (patients) [25]	Acceptance/hesitation of patients [25,26,32,37,46,47]
			Concerns (of patients) having fewer direct interactions [31]
			Negative emotional experiences of patients [49]
Implementation Process	Teaming	Interdisciplinary collaboration in teams [29,44,45,48,52]	
		Learning from each other [43]	
	Planning	Definition of clear service objectives, expectations and limits [33]	Unclear pathways, policies and procedures [27,33,46]
		Providers’ autonomy and flexibility in implementing TR into practice [47]	Changes in role and responsibilities/disruption in existing workflows [31]
		Well-planned implementation [38]	Lack of coordination among multidisciplinary clinicians [41]
			Uncertainty in the implementation process [26]
			Pre- and postimplementation support/Leadership support [36,41]
			High expectations (both sides) [26]
	Engaging	Consistent use [46]	Underuse and undervalue TR [42]
		Staff engagement [27]	Staff are more comfortable with face-to-face therapy [42]
		Innovation culture and enthusiasm among teams and organisations [29,46,52]	Resistance to change [39]
	Reflecting and Evaluation	Systematically sharing of lessons learned during implementation [46]	

## Data Availability

Not applicable.

## Supplementary Materials

The following supporting information can be downloaded via this link: https://www.mdpi.com/article/10.3390/healthcare12060619/s1, Table S1: Search string of Web of Science (Search date 18/05/2022, updated on 02/10/2023); Table S2: Search string of MEDLINE (Search date 18/05/2022, updated on 02/10/2023); Table S3: Search string of EMBASE (Search date 18/05/2022, updated on 02/10/2023); Table S4: Search string of Psyndex (Search date 18/05/2022, updated on 02/10/2023); Table S5: Study characteristics.

## References

[1] Valle C., Schmitt-Sody M. (2023). Digitalization in rehabilitation. Orthopadie.

[2] Turolla A., Rossettini G., Viceconti A., Palese A., Geri T. (2020). Musculoskeletal Physical Therapy during the COVID-19 Pandemic: Is Telerehabilitation the Answer?. Phys. Ther..

[3] Bayly J., Bradshaw A., Fettes L., Omarjee M., Talbot-Rice H., Walshe C., Sleeman K.E., Bajwah S., Dunleavy L., Hocaoglu M. (2022). Understanding the impact of the COVID-19 pandemic on delivery of rehabilitation in specialist palliative care services: An analysis of the CovPall-Rehab survey data. Palliat. Med..

[4] Meyding-Lamadé U., Bassa B., Tibitanzl P., Davtyan A., Lamadé E.K., Craemer E.M. (2021). Telerehabilitation: Von der virtuellen Welt zur Realität—Medizin im 21. Jahrhundert: Videogestützte Therapie in Zeiten von COVID-19. Der Nervenarzt.

[5] Fiani B., Siddiqi I., Lee S.C., Dhillon L. (2020). Telerehabilitation: Development, Application, and Need for Increased Usage in the COVID-19 Era for Patients with Spinal Pathology. Cureus.

[6] John M. (2017). Telemedizinische Assistenzsysteme in der Rehabilitation und Nachsorge—Projekte, Technologien und Funktionen. B&G.

[7] Brennan D., Tindall L., Theodoros D., Brown J., Campbell M., Christiana D., Smith D., Cason J., Lee A. (2010). A Blueprint for Telerehabilitation Guidelines. Int. J. Telerehabil..

[8] Latifi R. (2008). Telerehabilitation: Current Perspectives.

[9] Cottrell M.A., Galea O.A., O’Leary S.P., Hill A.J., Russell T.G. (2017). Real-time telerehabilitation for the treatment of musculoskeletal conditions is effective and comparable to standard practice: A systematic review and meta-analysis. Clin. Rehabil..

[10] Cox N.S., Dal Corso S., Hansen H., McDonald C.F., Hill C.J., Zanaboni P., Alison J.A., O’Halloran P., Macdonald H., Holland A.E. (2021). Telerehabilitation for chronic respiratory disease. Cochrane Database Syst. Rev..

[11] Velayati F., Ayatollahi H., Hemmat M. (2020). A Systematic Review of the Effectiveness of Telerehabilitation Interventions for Therapeutic Purposes in the Elderly. Methods Inf. Med..

[12] Carlbring P., Andersson G., Cuijpers P., Riper H., Hedman-Lagerlöf E. (2018). Internet-based vs. face-to-face cognitive behavior therapy for psychiatric and somatic disorders: An updated systematic review and meta-analysis. Cogn. Behav. Ther..

[13] Bican R., Christensen C., Fallieras K., Sagester G., O’Rourke S., Byars M., Tanner K. (2021). Rapid Implementation of Telerehabilitation for Pediatric Patients during COVID-19. Int. J. Telerehabil..

[14] Pearce L., Costa N., Sherrington C., Hassett L. (2023). Implementation of digital health interventions in rehabilitation: A scoping review. Clin. Rehabil..

[15] Damschroder L.J., Aron D.C., Keith R.E., Kirsh S.R., Alexander J.A., Lowery J.C. (2009). Fostering implementation of health services research findings into practice: A consolidated framework for advancing implementation science. Implement. Sci..

[16] Glegg S.M.N., Levac D.E. (2018). Barriers, Facilitators and Interventions to Support Virtual Reality Implementation in Rehabilitation: A Scoping Review. Phys. Med. Rehabil..

[17] Nizeyimana E., Joseph C., Plastow N., Dawood G., Louw Q.A. (2022). A scoping review of feasibility, cost, access to rehabilitation services and implementation of telerehabilitation: Implications for low- and middle-income countries. Digit. Health.

[18] Subedi N., Rawstorn J.C., Gao L., Koorts H., Maddison R. (2020). Implementation of Telerehabilitation Interventions for the Self-Management of Cardiovascular Disease: Systematic Review. JMIR Mhealth Uhealth.

[19] Rabanifar N., Abdi K. (2021). Barriers and Challenges of Implementing Telerehabilitation: A Systematic Review. IRJ.

[20] Stampa S., Thienel C., Tokgöz P., Razum O., Dockweiler C. OSF Registries. Protocol for a Scoping Review on Implementation Conditions of Telerehabilitation. https://osf.io/saqyu.

[21] Arksey H., O’Malley L. (2005). Scoping studies: Towards a methodological framework. Int. J. Soc. Res. Methodol..

[22] Von Elm E., Schreiber G., Haupt C.C. (2019). Methodische Anleitung für Scoping Reviews (JBI-Methodologie). Z. Evid. Fortbild. Qual. Gesundheitswes..

[23] Peters M.D., Godfrey C., McInerney P., Munn Z., Tricco A.C., Khalil H. (2020). Updated methodological guidance for the conduct of scoping reviews. JBI Evid. Synth..

[24] Tricco A.C., Lillie E., Zarin W., O’Brien K.K., Colquhoun H., Levac D., Moher D., Peters M.D.J., Horsley T., Weeks L. (2018). PRISMA Extension for Scoping Reviews (PRISMA-ScR): Checklist and Explanation. Ann. Intern. Med..

[25] Bairapareddy K.C., Alaparthi G.K., Jitendra R.S., Prathiksha, Rao P.P., Shetty V., Chrasekaran B. (2021). We are so close; yet too far: Perceived barriers to smartphone-based telerehabilitation among healthcare providers and patients with Chronic Obstructive Pulmonary Disease in India. Heliyon.

[26] Rabanifar N., Hoseini M.A., Abdi K. (2022). Exploring Barriers to Implementing Telerehabilitation from experiences of managers, policymakers, and providers of rehabilitation services in Iran: A Qualitative Study. Med. J. Islam. Repub. Iran.

[27] Teriö M., Eriksson G., Kamwesiga J.T., Guidetti S. (2019). What’s in it for me? A process evaluation of the implementation of a mobile phone-supported intervention after stroke in Uganda. BMC Public Health.

[28] The World Bank The World by Income and Region. https://datatopics.worldbank.org/world-development-indicators/the-world-by-income-and-region.html.

[29] Auger L.-P., Moreau E., Côté O., Guerrera R., Rochette A., Kairy D. (2023). Implementation of Telerehabilitation in an Early Supported Discharge Stroke Rehabilitation Program before and during COVID-19: An Exploration of Influencing Factors. Disabilities.

[30] Banerjee-Guenette P., Bigford S., Glegg S.M.N. (2020). Facilitating the Implementation of Virtual Reality-Based Therapies in Pediatric Rehabilitation. Phys. Occup. Ther. Pediatr..

[31] Caughlin S., Mehta S., Corriveau H., Eng J.J., Eskes G., Kairy D., Meltzer J., Sakakibara B.M., Teasell R. (2020). Implementing Telerehabilitation After Stroke: Lessons Learned from Canadian Trials. Telemed. J. E-Health.

[32] Farzad M., MacDermid J., Ferreira L., Szekeres M., Cuypers S., Shafiee E. (2023). A description of the barriers, facilitators, and experiences of hand therapists in providing remote (tele) rehabilitation: An interpretive description approach. J. Hand Ther..

[33] Hurtubise K., Pratte G., Hamel C., Clapperton I., Camden C. (2022). Rethinking early intervention rehabilitation services for children with motor difficulties: Engaging stakeholders in the conceptualization of telerehabilitation primary care. Disabil. Rehabil..

[34] Jiancaro T., Bayoumi A.M., Ibáñez-Carrasco F., Torres B., McDuff K., Brown D.A., Chan Carusone S., Tang A., Loutfy M., Cobbing S. (2023). Factors influencing initial implementation of an online community-based exercise intervention with adults living with HIV: A systems approach. Front. Rehabil. Sci..

[35] Kairy D., Lehoux P., Vincent C. (2014). Exploring routine use of telemedicine through a case study in rehabilitation. Rev. Panam. Salud Publica..

[36] Munce S., Andreoli A., Bayley M., Guo M., Inness E.L., Kua A., McIntyre M. (2023). Clinicians’ Experiences of Implementing a Telerehabilitation Toolkit During the COVID-19 Pandemic: Qualitative Descriptive Study. JMIR Rehabil. Assist. Technol..

[37] Pol M., Qadeer A., van Hartingsveldt M., Choukou M.-A. (2023). Perspectives of Rehabilitation Professionals on Implementing a Validated Home Telerehabilitation Intervention for Older Adults in Geriatric Rehabilitation: Multisite Focus Group Study. JMIR Rehabil. Assist. Technol..

[38] Yang C.-L., Waterson S., Eng J.J. (2021). Implementation and Evaluation of the Virtual Graded Repetitive Arm Supplementary Program (GRASP) for Individuals with Stroke during the COVID-19 Pandemic and Beyond. Phys. Ther..

[39] Cottrell M.A., Hill A.J., O’Leary S.P., Raymer M.E., Russell T.G. (2017). Service provider perceptions of telerehabilitation as an additional service delivery option within an Australian neurosurgical and orthopaedic physiotherapy screening clinic: A qualitative study. Musculoskelet. Sci. Pract..

[40] Cox N.S., Scrivener K., Holl A.E., Jolliffe L., Wighton A., Nelson S., McCredie L., Lannin N.A. (2021). A Brief Intervention to Support Implementation of Telerehabilitation by Community Rehabilitation Services During COVID-19: A Feasibility Study. Arch. Phys. Med. Rehabil..

[41] Lau A.Y., Piper K., Bokor D., Martin P., Lau V.S., Coiera E. (2017). Challenges During Implementation of a Patient-Facing Mobile App for Surgical Rehabilitation: Feasibility Study. JMIR Hum. Factors.

[42] Pitt R., Hill A.J., Theodoros D., Russell T. (2018). “I definitely think it’s a feasible and worthwhile option”: Perspectives of speech-language pathologists providing online aphasia group therapy. Aphasiology.

[43] Ross M.H., Nelson M., Parravicini V., Weight M., Tyrrell R., Hartley N., Russell T. (2023). Staff perspectives on the key elements to successful rapid uptake of telerehabilitation in medium-sized public hospital physiotherapy departments. Physiother. Res. Int..

[44] Ahonle Z.J., Kreider C.M., Hale-Gallardo J., Castaneda G., Findley K., Ottomanelli L., Romero S. (2021). Implementation and use of video tele-technologies in delivery of individualized community-based vocational rehabilitation services to rural veterans. J. Vocat. Rehabil..

[45] Duran A.T., Keener-DeNoia A., Stavrolakes K., Fraser A., Blanco L.V., Fleisch E., Pieszchata N., Cannone D., Keys McKay C., Whittman E. (2023). Applying User-Centered Design and Implementation Science to the Early-Stage Development of a Telehealth-Enhanced Hybrid Cardiac Rehabilitation Program: Quality Improvement Study. JMIR Form. Res..

[46] Gorzelitz J.S., Bouji N., Stout N.L. (2022). Program Barriers and Facilitators in Virtual Cancer Exercise Implementation: A Qualitative Analysis. Transl. J. Am. Coll. Sports Med..

[47] Hale-Gallardo J.L., Kreider C.M., Jia H., Castaneda G., Freytes I.M., Ripley D.C.C., Ahonle Z.J., Findley K., Romero S. (2020). Telerehabilitation for rural veterans: A qualitative assessment of barriers and facilitators to implementation. J. Multidiscip. Healthc..

[48] Damhus C.S., Emme C., Hansen H. (2018). Barriers and enablers of COPD telerehabilitation—A frontline staff perspective. Int. J. Chron. Obstruct. Pulmon. Dis..

[49] Ferreira-Correia A., Barberis T., Msimanga L. (2018). Barriers to the implementation of a computer-based rehabilitation programme in two public psychiatric settings. S. Afr. J. Psychiatr..

[50] Aloyuni S., Alharbi R., Kashoo F., Alqahtani M., Alanazi A., Alzhrani M., Ahmad M. (2020). Knowledge, Attitude, and Barriers to Telerehabilitation-Based Physical Therapy Practice in Saudi Arabia. Healthcare.

[51] Brouns B., van Bodegom-Vos L., de Kloet A.J., Vlieland Vliet T.P., Gil I.L.C., Souza L.M.N., Braga L.W., Meesters J.J.L. (2020). Differences in factors influencing the use of eRehabilitation after stroke; a cross-sectional comparison between Brazilian and Dutch healthcare professionals. BMC Health Serv. Res..

[52] Kraaijkamp J.J.M., Persoon A., Aurelian S., Bachmann S., Cameron I.D., Choukou M.-A., Dockery F., Eruslanova K., Gordon A.L., Grund S. (2023). eHealth in Geriatric Rehabilitation: An International Survey of the Experiences and Needs of Healthcare Professionals. J. Clin. Med..

[53] Yosef A.B., Maeir T., Khalailh F., Gilboa Y. (2022). Perceived feasibility of an occupation-based telerehabilitation intervention for older adults with chronic health conditions in Israel. Hong Kong J. Occup. Ther..

[54] Damschroder L.J., Reardon C.M., Opra Widerquist M.A., Lowery J. (2022). Conceptualizing outcomes for use with the Consolidated Framework for Implementation Research (CFIR): The CFIR Outcomes Addendum. Implement. Sci..

[55] Bouabida K., Lebouché B., Pomey M.-P. (2022). Telehealth and COVID-19 Pandemic: An Overview of the Telehealth Use, Advantages, Challenges, and Opportunities during COVID-19 Pandemic. Healthcare.

[56] Bassa B., Hahner F., Braun S., Meyding-Lamadé U. (2024). Telemedizin und internationale Projekte: Von Asien nach Afrika—Chancen der Zukunft?. Der Nervenarzt.

