# Supplementary material for: Factors Facilitating and Inhibiting the Implementation of Telerehabilitation—A Scoping Review
Source: Healthcare (Basel). 2024 Mar 8;12(6):619. doi: 10.3390/healthcare12060619 (PMC10970368; doi:10.3390/healthcare12060619)
Supplement: Supplementary file 1 [file healthcare-12-00619-s001.zip › healthcare-2885247-supplementary.pdf]

Supplementary Materials

Search strings

Table S1: Search string of Web of Science (search date 18/05/2022, updated on 02/10/2023).

Number	Type of search term	Search term
#1	TS	telerehab*
#2	TS	tele-rehab*
#3	TS	erehab*
#4	TS	e-rehab*
#5	TS	virtual* NEAR/5 rehab*
#6	TS	remote* NEAR/5 rehab*
#7	TS	digital* NEAR/5 rehab*
#8	TS	online NEAR/5 rehab*
#9	TS	mobile NEAR/5 rehab*
#10	TS	web-based NEAR/5 rehab*
#11	TS	computer-based NEAR/5 rehab*
#12	TS	internet-based NEAR/5 rehab*
#13	**#1 OR #2 OR #3 OR #4 OR #5 OR #6 OR #7 OR #8 OR #9 OR #10 OR #11 OR #12**	
#14	TS	influenc*
#15	TS	barrier*
#16	TS	inhibit*
#17	TS	imped*
#18	TS	hinder*
#19	TS	hindrance*
#20	TS	facilitat*
#21	TS	promot*
#22	**#14 OR#15 OR #16 OR #17 OR #18 OR #19 OR #20 OR #21**	
#23	TS	diffusion of innovation
#24	TS	implement*
#25	TS	adopt*
#26	TS	realis*
#27	TS	realiz*
#28	**#23 OR#24 OR #25 OR #26 OR #27**	
#29	**#13 AND #22 AND #28<br>AND (LA="GERMAN" OR "ENGLISH")<br>AND (Timespan: 2012-05-18 to 2023-10-02)**	

Table S2: Search string of MEDLINE (Search date 18/05/2022, updated on 02/10/2023).

Number	Type of search term	Search term
#1	MeSH	telerehabilitation
#2	Titel/Abstract	telerehab*
#3	Titel/Abstract	tele-rehab*
#4	Titel/Abstract	erehab*
#5	Titel/Abstract	e-rehab*
#6	**#1 OR #2 OR #3 OR #4 OR #5**	
#7	Titel/Abstract	virtual*
#8	Titel/Abstract	remote*
#9	Titel/Abstract	digital*
#10	Titel/Abstract	online
#11	Titel/Abstract	mobile
#12	Titel/Abstract	web-based
#13	Titel/Abstract	computer-based
#14	Titel/Abstract	internet-based
#15	Titel/Abstract	rehab*
#16	**(#7 OR #8 OR #9 OR #10 OR #11 OR #12 OR #14) AND #15**	
#17	**#6 OR #16**	
#18	Titel/Abstract	influenc*
#19	Titel/Abstract	barrier*
#20	Titel/Abstract	inhibit*
#21	Titel/Abstract	imped*
#22	Titel/Abstract	hinder*
#23	Titel/Abstract	hindrance*
#24	Titel/Abstract	facilitat*
#25	Titel/Abstract	promot*
#26	**#18 OR #19 OR #20 OR #21 OR #22 OR #23 OR #24 OR #25**	
#27	MeSH	diffusion of innovation
#28	Titel/Abstract	diffusion of innovation
#29	Titel/Abstract	implement*
#30	Titel/Abstract	adopt*
#31	Titel/Abstract	realis*
#32	Titel/Abstract	realiz*
#33	**#27 OR #28 OR #29 OR #30 OR #31 OR #32**	
#34	**(#17 AND #26 AND #33)<br>AND ((y_10[Filter]) AND (english[Filter] OR german[Filter]))**	

Table S3: Search string of Embase (Search date 18/05/2022, updated on 02/10/2023).

Number	Type of search term	Search term
#1	exp	telerehabilitation
#2	ab,ti	tele rehab*
#3	ab,ti	telerehab*
#4	ab,ti	erehab*
#5	ab,ti	e-rehab*
#6	ab,ti	virtual* NEXT/5 rehab*
#7	ab,ti	remote* NEXT/5 rehab*
#8	ab,ti	digital* NEXT/5 rehab*
#9	ab,ti	online NEXT/5 rehab*
#10	ab,ti	mobile NEXT/5 rehab*
#11	ab,ti	web based NEXT/5 rehab*
#12	ab,ti	computer based NEXT/5 rehab*
#13	ab,ti	internet based NEXT/5 rehab*
#14	**#1 OR #2 OR #3 OR #4 OR #5 OR #6 OR #7 OR #8 OR #9 OR #10 OR #11 OR #12 OR #13**	
#15	ab,ti	influenc*
#16	ab,ti	barrier*
#17	ab,ti	inhibit*
#18	ab,ti	imped*
#19	ab,ti	hinder*
#20	ab,ti	hindrance*
#21	ab,ti	facilitat*
#22	ab,ti	promot*
#23	**#15 OR #16 OR #17 OR #18 OR #19 OR #20 #21 OR #22**	
#24	exp	diffusion of innovation
#25	ab,ti	diffusion of innovation
#26	ab,ti	implement*
#27	ab,ti	adopt*
#28	ab,ti	realis*
#29	ab,ti	realiz*
#30	**#24 OR #25 OR #26 OR #27 OR #28 OR #29**	
#31	**(#14 AND #23 AND #30) AND ([english]/lim OR [german]/lim) AND [2012-2023]/py**	

Table S4. Search string of Psyndex (Search date 18/05/2022, updated on 02/10/2023).

Number	Search term
#1	telerehab*
#2	tele-rehabilitation
#3	e-rehabilitation
#4	eRehab*
#5	virtual* N5 rehab*
#6	remote* N5 rehab*
#7	digital* N5 rehab*
#8	online N5 rehab*
#9	mobile N5 rehab*
#10	web-based N5 rehab*
#11	computer-based N5 rehab*
#12	internet-based N5 rehab*
#13	**#1 OR #2 OR #3 OR #4 OR #5 OR #6 OR #7 OR #8 OR #9 OR #10 OR #11 OR #12**
#14	influenc*
#15	barrier*
#16	inhibit*
#17	imped*
#18	hinder*
#19	hindrance*
#20	facilitat*
#21	promot*
#22	**#14 OR#15 OR #16 OR #17 OR #18 OR #19 OR #20 OR #21**
#23	diffusion of innovation
#24	implement*
#25	adopt*
#26	realis*
#27	realiz*
#28	**#23 OR#24 OR #25 OR #26 OR #27**
#29	**(#13 AND #22 AND #28) AND (LA=deu OR LA=eng) AND (PY>=2012 AND PY<=2023)**

Table S5. Study characteristics.

First author, year,	Country	Study design	Study population (patients)	Study population (health professionals)	Diagnosis group	Type of TR	Status of implementation / experiences	Implementation framework
**Ahonle (2021) [44]**	USA	Qualitative design: semi-structured interviews (cross-sectional)	N/A	Rehabilitation specialists (n = 3)	Various disabilities	Teleconsultation, community-based rehabilitation for veterans (video-based)	After implementation in routine care	N/A
**Aloyuni (2020) [50]**	Saudi Arabia	Quantitative design: online-survey (cross-sectional)	N/A	PTs (n = 347)	Various diagnostic groups	Image-based TR (videoconferences), sensor-based TR (tilt switches, gyroscope, accelerometers), VR	19.9% of respondents use TR at work	N/A
**Auger (2023) [29]**	Canada	Descriptive qualitative design: focus group, semi-structured interviews were conducted after the implementation phase (cross-sectional)	N/A	OTs, social workers, SLPs, PTs, others (n = 9)	Stroke	TR in post-stroke, videoconference	During implementation	CFIR
**Bairapareddy (2021) [25]**	India	Quantitative study (feasibility study): online survey (cross-sectional)	COPD patients (n = 30)	Pulmonary care physicians, rehabilitation nurses, PTs (n = 52)	COPD	Smartphone-based TR for COPD management	Before implementation in routine care (A few HPs (n = 4) already implemented TR; patients had a minimum level of experience with TR)	N/A
**Banerjee-Guenette (2019) [30]**	Canada	Mixed-methods-design: questionnaires, tracking of VR-usage	N/A	PTs, OTs, one rehabilitation assistant (n = 11)	Various paediatric diseases	Variety of VR/AVR, e.g., Nintendo Wii© and WiiFit©	Before implementation	DTBT

Table S5. Study characteristics.

First author, year,	Country	Study design	Study population (patients)	Study population (health professionals)	Diagnosis group	Type of TR	Status of implementation / experiences	Implementation framework
		(pre- and post-follow-up)						
**Brouns 2020 [51]**	Netherlands	Quantitative study: online-survey (cross-sectional)	N/A	BHP (n = 99), DHP (n = 105)	Stroke	eRehabilitation: e.g. physical and cognitive exercises, education and communication, not further described	44% with and 66% without experiences	Framework of Grol
**Caughlin et al. (2019) 31**	Canada	Summary of six studies**:** (feasibility, pre and post-design, pilot trials, randomised controlled trials (RCTs), and observational studies)	Stroke patients: 1) (n = 44) 2) (n = 11) 3) (n = 7) 4) (n = 126) 5) (n = 74) 6) (n = 51)	2) PTs, OTs, SLPs, managers, technical support: (n = 11)	Stroke	1) to 5): Different remote services for stroke patients, e. g., speech therapy, memory training, self-management, (e. g. via videoconference) 6) VR for upper limb training	Before implementation or in an early phase of implementation in routine care.	General model of stroke rehabilitation
**Cottrell (2017) [39]**	Australia	Qualitative descriptive study: semi-structured interviews (cross-sectional)	N/A	PTs (n = 26)	Chronic musculoskeletal diseases	TR in general	Before implementation in routine care	N/A
**Cox (2017) [40]**	Australia	Quantitative study (implementation study**):** online-survey (pre-and post-design)	N/A	PTs, OTs (n = 31 to 26)	Neurologic, ageing or musculoskeletal disabilities:	Group education (videoconferences) for children and adults	During implementation in routine care	TDF
**Damhus et al. (2018) [48]**	Denmark	Qualitative design: semi-structured interviews, focus	N/A	HPs (n = 25)	COPD	Online-exercise sessions to patients with COPD, real-time video	With experiences (n = 4 focus groups), without experiences, (n = 2	TDF

Table S5. Study characteristics.

First author, year,	Country	Study design	Study population (patients)	Study population (health professionals)	Diagnosis group	Type of TR	Status of implementation / experiences	Implementation framework
		groups (cross-sectional)				telemedical technology	focus groups, n = 3 interviews)	
**Duran 2023 [45]**	USA	Qualitative design: (improvement study): interviews, design workshops, usability testing (user-centred-design)	CR patients (n=3)	Provider- and system-level stakeholders (n = 9) design workshops (n = 20), clinicians (n = 2)	Cardiac diseases	Development of a TR-prototype: (e.g. telemonitoring, educational videos); hybrid	Before implementation in routine care	TDF and CFIR
**Farzad (2023) [32]**	Canada	Qualitative descriptive study: semi-structured interviews (cross-sectional)	N/A	PTs, OTs (n = 14)	Hand diseases	Online hand therapy in general (video-conference, videos)	With experiences in providing TR	N/A
**Ferreira-Correia (2018) [49]**	South Africa	Qualitative descriptive design: semi-structured interviews, field notes (pre-and post-design)/a TR intervention was carried out	Patients with HIV (n = 4), patients with schizophrenia (n = 5)	N/A	HIV, schizophrenia	CogMed Working Memory Training Programme	Before implementation in routine care	N/A
**Gorzelitz (2022) [46]**	USA	Qualitative design: semi-structured interviews (cross-sectional)	N/A	Program-representatives (n = 24)	Cancer	Different TR exercise programmes (video-based)	After implementation in routine care	CFIR
**Hale-Gallardo (2020) [47]**	USA	Qualitative design: semi-structured interviews (pre-and post-design)	N/A	Programme managers and medical directors (n = 10)	Various disabilities	National TR programme for veterans, video technology	During first 18 month of implementation	RE-AIM framework
**Hurtubise (2022) [33]**	Canada	Qualitative design: focus groups, interviews (participatory research design)	Parents of children with motor difficulties (n=4)	Clinicians (n = 9), health care managers (n = 5) IT-consultants (n = 2)	Motor difficulties (children)	TR platform for children, not yet developed	Before implementation in routine care	A logic model for a new primary care TR intervention was developed

Table S5. Study characteristics.

First author, year,	Country	Study design	Study population (patients)	Study population (health professionals)	Diagnosis group	Type of TR	Status of implementation / experiences	Implementation framework
**Jiancaro (2023) [34]**	Canada	Qualitative descriptive study: communication with patients and team, in-person meetings, documents (cognitive work analysis)	Patients with HIV (n = 33)	Members of the implementation team (n = 10)	HIV	Tele-coaching exercise, online exercise class, wireless physical activity monitor (e. g. via videoconference)	During initial implementation phase	Cognitive work analysis approach
**Kairy (2014) [35]**	Canada	Qualitative design: focus groups, semi-structured interviews, project documents (case study)	Patients (n = 5), family members (n = 5)	HPs (n = 16), managers (n = 8)	Traumatic brain injuries, spinal cord injuries	Interprofessional care plans with HPs and patients, TR services (videoconference), patient education	Particularly successful incorporated into routine care	Giddens' theory of structuration
**Kraaijkamp (2023) [52]**	Switzerland	Quantitative study: multicentre online survey (cross- sectional)	N/A	HPs from 16 countries (n = 513)	Geriatric diseases	Different technologies, most apps and video conferencing	>50 percent had experience with eHealth, 20% integrated eHealth in daily practice	N/A
**Lau (2017) [41]**	Australia	Mixed-methods design (feasibility study): observation reports, issues log, emails, interviews; a TR intervention was carried out (case study)	Patients after a rotator cuff surgery (n = 9)	Clinicians (n = not mentioned)	Orthopaedic disorders	Patient-facing app using the Healthy.me platform, exercise videos	Before implementation in routine care	N/A
**Munce et al. (2023) [36]**	Canada	Qualitative descriptive design: interviews, focus groups (cross-sectional)	N/A	Physicians, OTs, PTs (n = 22)	Neurological and cardiac disorders, long-term care	Toronto Rehab Telerehab Toolkit; TR technology not further described	After implementation in routine care	N/A
**Pitt (2018) [42]**	Australia	Qualitative design (feasibility study): semi- structured interviews were conducted after the	N/A	SLPs (n = 3)	Aphasia	TR Group Aphasia Intervention and Networking (TeleGAIN) (via videoconference)	Before implementation in routine care, different	N/A

Table S5. Study characteristics.

First author, year,	Country	Study design	Study population (patients)	Study population (health professionals)	Diagnosis group	Type of TR	Status of implementation / experiences	Implementation framework
		intervention phase (cross-sectional) /					experiences with TR	
**Pol (2023) [37]**	Canada	Qualitative study: (exploratory study): focus groups (cross-sectional)	N/A	Rehabilitation professionals from (a) Netherlands (n = 5) and (b) Canada (n = 9)	Neurological and orthopaedic disorders, geriatric rehabilitation	Evidence-based sensor monitoring intervention (SMI) for older adults	Group (a) with experience, group (b) no experience, but introduced	MRC
**Rabanifar (2022) [26]**	Iran	Qualitative design: semi- structured interviews (cross-sectional)	N/A	Managers, policy makers and service providers (n = 26)	Different diagnoses	TR in general	All with TR background (experiences in receiving or providing TR)	N/A
**Ross (2022) [43]**	Australia	Qualitative design: semi- structured interviews (cross-sectional)	N/A	Physiotherapists (n = 25)	Musculoskeletal diseases, paediatric diseases, pelvic diseases	TR in general	After implementation in routine care	N/A
**Teriö et al. (2019) [27]**	Uganda	Mixed methods design (feasibility study): Semi-structured interviews, quantitative process data (single-case)	N/A	HPs (n = 12)	Stroke	Mobile phone-supported and family-centred rehabilitation intervention to increase daily activities	Before implementation in routine care	i-PARIHS framework
**Yang et al. (2021) [38]**	Canada	Mixed-methods-design: questionnaires, additional data, (pre-and post-design)	Patients with stroke (n = 9)	N/A	Stroke	GRASP for individuals with stroke—video-conference software	During implementation, the organisation continued to offer the programme	RE-AIMframework
**Yosef (2022) [53]**	Israel	Mixed-methods-design (feasibility study): focus groups, questionnaire (cross-sectional)	N/A	OTs (n = 10)	Chronic health conditions: neurological and orthopaedic disorders (older adults)	In-home TR service for older adults with chronic health conditions (videoconference)	Before Implementation in routine care	N/A

*N/A: not applicable, TR: Telerehabilitation, VR: Virtual Reality, AVR: Active Video Gaming, COPD: Chronic Obstructive Pulmonary Disease, CR: Cardiac Rehabilitation, PT: Physiotherapists, SLPs: Speech-Language Pathologists, OTs: Occupational Therapists, HPs: Health Professionals, BHP: Brazilian Healthcare Professionals, DHP: Dutch Healthcare Professionals, CFIR: Consolidated Framework

for Implementation research, RE-AIM: Reach, Effectiveness, Adoption, Implementation, Maintenance Framework, MRC: Medical Research Council Framework, i-PARIHS: The Promoting Action on Research Implementation in Health Services Framework, TDF: The Theoretical Domains Framework, DTPB: Decomposed Theory of Planned Behavior, GRASP: Graded Repetitive Arm Supplementary Programme
